# The Pathophysiology and Treatment of Essential Tremor: The Role of Adenosine and Dopamine Receptors in Animal Models

**DOI:** 10.3390/biom11121813

**Published:** 2021-12-02

**Authors:** Barbara Kosmowska, Jadwiga Wardas

**Affiliations:** Department of Neuropsychopharmacology, Maj Institute of Pharmacology Polish Academy of Sciences, 31-343 Kraków, Poland; mroz@if-pan.krakow.pl

**Keywords:** adenosine, adenosine A1 receptors, animal models, dopamine D3 receptors, essential tremor, glutamate, harmaline, pathomechanism, thalamus

## Abstract

Essential tremor (ET) is one of the most common neurological disorders that often affects people in the prime of their lives, leading to a significant reduction in their quality of life, gradually making them unable to independently perform the simplest activities. Here we show that current ET pharmacotherapy often does not sufficiently alleviate disease symptoms and is completely ineffective in more than 30% of patients. At present, deep brain stimulation of the motor thalamus is the most effective ET treatment. However, like any brain surgery, it can cause many undesirable side effects; thus, it is only performed in patients with an advanced disease who are not responsive to drugs. Therefore, it seems extremely important to look for new strategies for treating ET. The purpose of this review is to summarize the current knowledge on the pathomechanism of ET based on studies in animal models of the disease, as well as to present and discuss the results of research available to date on various substances affecting dopamine (mainly D3) or adenosine A1 receptors, which, due to their ability to modulate harmaline-induced tremor, may provide the basis for the development of new potential therapies for ET in the future.

## 1. Introduction

Tremor is an involuntary, rhythmic, and oscillating movement produced by alternating or synchronous contractions of antagonistic muscles in different parts of the body. Tremor can be physiological or pathological. Physiological tremor is a low-amplitude, high-frequency tremor that is invisible or occasional, in contrast to pathological tremor, which is usually clearly visible and persists for an extended period of time [1,2]. Physiological tremor can also be enhanced (the so-called increased physiological tremor) under the influence of anxiety, stress, certain medications, and excessive caffeine consumption, and these behaviors generally do not require pharmacotherapy and disappear after the elimination of the causative agent [3,4]. Tremor can also be classified according to the circumstances and conditions under which they occur: (1) resting tremor occurs in the resting state of the limb, which does not have to counteract the force of gravity (parkinsonian-type tremor); (2) action tremor, which can appear when the patient voluntarily holds a part of the body in a selected position, against the force of gravity (postural tremor, which can depend, or be independent of, a specific posture or position of a limb); (3) kinetic tremor, which appears at the time of performing a specific voluntary movement; and (4) isometric tremor, which appears during the isometric contraction of the muscles against constant resistance, e.g., squeezing a selected object in the hand [1,3]. In clinical practice, other characteristics, such as frequency and amplitude, are also essential for the assessment and diagnosis of tremor. The frequency range for most pathological tremors is between 4–12 Hz, except for tremors resulting from damage to the cerebellum or brainstem, which are usually of a lower frequency and are characterized by a frequency below 4 Hz [5].

## 2. Essential Tremor (ET)

Essential tremor (ET) is a pathological tremor. The familial ET was first described by the German physician Georg Friedrich Most in 1836, while the term essential tremor (originally from the Italian language ‘tremore semplice essenziale’) was used for the first time in 1874 by the professor of medicine Pietro Burresi, who described the case of an 18-year-old man suffering from severe hand tremor while moving, as well as head tremor [6]. In 1817, James Parkinson was the first to point out that ET was a disorder separate from Parkinson’s disease (PD), but his report was not published until 1887. A classic description of the disease was presented in 1925 by the Russian neurologist Lazar Salomonowicz Minor, after which ET is often also called ‘Minor’s disease’ [7,8]. 

ET is a chronic and progressive neurological disease whose main symptom is tremor, classified as postural or kinetic, with a frequency ranging from 4 to 12 Hz. Tremor in this disease affects various parts of the body, mainly the hands (approximately 95% of patients), head (at minimum, 34%), tongue (about 30%), legs (approximately 30%), and, less frequently, the voice (at minimum, 12%), face/chin (about 7%), and torso (approximately 5%) [7]. As the patient ages and the disease becomes more severe, a gradual reduction in the frequency of tremor and an increase in its amplitude are observed. Therefore, in the elderly, the frequency of tremor may be similar to that of tremor in PD. Although the frequency of tremor is relatively constant in a particular patient (apart from its reduction as the disease progresses), the amplitude may fluctuate in various ways, e.g., as a result of attention focus or distraction [2]. 

ET has long been viewed as a monosymptomatic disease with tremor as the only characteristic symptom. Today, according to the latest literature, in addition to isolated ET, there is also ‘ET plus’ and ‘ET-PD’. If patients exhibit a cognitive impairment (determined by the appropriate tests) such as tandem gait abnormalities, ataxia, dystonia, or resting tremor, they are diagnosed as ‘ET plus’. Furthermore, if patients meet the diagnostic criteria for both ET and PD, the syndrome is called ‘ET-PD’ [9]. In recent years, the non-motor symptoms that may accompany ET have been receiving increasing attention. Among them, the following can be distinguished: (1) cognitive features (including a complete spectrum from mild cognitive difficulty to dementia), psychiatric features (depression, apathy, anxiety, and personal characteristics), sensory features (hearing or olfactory abnormalities), and other non-motor characteristics (e.g., sleep dysregulation) [9]. According to a study by Sinoff and Badarny [10], approximately 70% of patients with ET show a mild cognitive impairment (MCI), the most common symptoms of which include speech fluidity, verbal memory disorders, and concentration disturbances. Furthermore, a large proportion of patients also suffer from anxiety (25%) or depression (about 20%). The nature of these two psychiatric symptoms in ET remains unclear. On the one hand, they may occur as a consequence of motor dysfunction secondary to tremor, which, through a negative impact on the performance of the basic functions of everyday life, leads to a decrease in the patient’s independence, lower self-esteem, and social isolation, and consequently to the development of depression or anxiety. They may also be due to the patient’s pharmacotherapy (a side effect of medications) or as a result of neurodegeneration in various parts of the nervous system, assuming that ET is a neurodegenerative disease [11]. However, the results of studies by Louis et al. [12] indicated that in patients who self-reported depression compared to those taking antidepressants, the incidence of ET was much higher, suggesting that depression may be the primary symptom of the disease development and may constitute a kind of pre-motor marker or risk factor for ET. 

### 2.1. Epidemiology

ET is one of the most common movement disorders and is the most common form of tremor. The incidence of ET is assessed as very variable depending on the studied population. A summary of the epidemiological studies carried out to date can be found in the recent meta-analysis conducted by Louis and McCreary [13], based on 42 population studies that came from 23 different countries on six continents (Asia, Europe, North and South America, Africa, and Australia). The analysis shows that the prevalence of ET in the general population among all ages is 1.33%. The prevalence increases markedly with age, as ET affects from 0.5 to 26.1% of people over 65 years of age (mean = 6.9%; median = 5.9%) and from 1.2 to 42.9% (mean = 11.4%; median = 9.3%) of people over 80. Interestingly, ET has a bimodal distribution in the second and sixth decades of life [14]. 

### 2.2. ET Etiology

There are two forms of ET, familial (hereditary) and sporadic (non-hereditary). It is worth noting that according to the study conducted on a group of 195 patients with ET, the age at the onset of tremor is much lower for the familial (40.7 years) than for the sporadic form (57.7) [15].

#### 2.2.1. Genetic Background

The significant role of genetic factors in the etiology of ET is supported by the high prevalence of a positive family history of tremor (20–90%) in patients with ET [16], the appearance of the genetic anticipation phenomenon (tremor appears at an earlier age in the next generation) [17], and higher concordance rates of ET for monozygotic twins (0.60–0.93) than for dizygotic twins (0.27–0.29) [18,19]. In the 1940s, studies on members of families with ET showed that the disease is inherited in an autosomal dominant mode [20]. Today, we know that ET inheritance is more complex and could also have an autosomal recessive, X-linked, or non-Mendelian pattern [17]. 

A genetic linkage analysis identified four loci within which genes related to ET could be located. These areas are sited on chromosome 3q13 (ETM1) [21], 2p25–p22 (ETM2) [22], 6p23 (ETM3) [23], and 5q35 (no specific name) [24]. In the ETM1 region (3q13.3), the gene that encodes the dopamine receptor D3 (*DRD3*) can be found. An initial study has shown that the Ser9Gly variant of the *DRD3* gene (*DRD3-Gly*) could be associated with the risk of ET [25]. The pathophysiological implications of this variant are of interest. Dopamine D3 receptors are involved, among others, in the regulation of Purkinje cell (PC) excitability in the cerebellar cortex, i.e., the structure involved in the pathomechanism of tremor. In vitro studies have also shown that dopamine has a four- to five-times greater affinity for the receptor encoded by the *DRD3-Gly* variant than for the unmutated form; therefore, it was suggested that *DRD3-Gly* may contribute to the excessive inhibition of GABAergic PC activity [25]. Unfortunately, the conclusion of the large meta-analysis of ET genetics is that it is unlikely that the ETM1 region contains the causative gene for ET, or that *DRD3* polymorphism is a major risk factor for ET [17,26].

Genome-wide association studies (GWAS) among patients with ET have shown a relationship between several variants in *LINGO1*, *SLC1A2*, *STK32B*, *PPARGC1A*, and *CTNNA3* genes and ET, but none of them have been confirmed in replication studies. Furthermore, case-control association studies for candidate variants have not significantly linked any gene with the risk of developing the disease. Exome studies described the association of several genes with the familial form of ET (*FUS*, *HTRA2*, *TENM4*, *SORT1*, *SCN11A*, *NOTCH2NLC*, *NOS3*, *KCNS2*, *HAPLN4*, *USP46*, *CACNA1G*, *SLIT3*, *CCDC183*, *MMP10,* and *GPR151*), but they were found only in individual families, suggesting that they were some kind of private polymorphism. Therefore, the identification of the genes responsible for ET requires further research [17].

#### 2.2.2. Environmental Factors

The large percentage of familial forms of ET indicate the importance of the genetic basis of this disease; however, due to the presence of the sporadic form as well, it seems that environmental factors may also have a significant influence on the development of ET. Chemical compounds that occur in the surrounding environment and are suspected to be involved in the pathogenesis of ET include organochlorine pesticides (OCPs), as prolonged exposure to them can result in action tremor with a frequency of 6–8 Hz [27]. Studies in animals indicate that OCPs can produce some pathological changes in the cerebellar cortex and can decrease the extracellular level of GABA as a result of the GABA-A receptor blockade [27]. Other substances that can cause tremor are both the organic and inorganic compounds of lead and mercury. In animal studies, lead has been shown to be toxic to the cerebellum, as perinatal exposure to this element can cause the degeneration of cerebellar PCs [27]. Another group of compounds capable of inducing tremor that occur naturally in the environment are β-carboline alkaloids (βCAs), which are structural analogs of MPTP (1-methyl-4-phenyl-1,2,3,6-tetrahydropyridine) and MPP+ (1-methyl-4-phenylpyridine), neurotoxins that cause symptoms of PD. Like MPTP, some βCAs are highly neurotoxic and induce intense and generalized ET-like tremor in laboratory animals (mice, rats, and monkeys) [8]. The best-known βCAs with high tremorogenic properties include harman, harmine, and harmaline. Harman is present in significant concentrations (ng/g) in meat that has been cooked for a long time, as well as in alcoholic beverages, tobacco smoke, coffee, some plants, algae, bacteria, and fungi. Importantly, βCA can also be produced endogenously in the brain or peripheral tissues by the Mannich reaction, using precursors such as tryptamine, tryptophan, serotonin, acetic aldehyde, and pyruvate [8,27]. Interestingly, studies conducted among ET patients indicated an increased level of harman in peripheral blood compared to healthy controls [28]. Studies in a small number of patients with ET also indicated an inverse correlation between blood harman levels and the NAA/tCr (N-acetylaspartate/total creatinine, an in vivo marker of neuronal viability) ratio in the cerebellum, suggesting that harman levels may be related to the dysfunction of this structure [29]. Postmortem studies have confirmed this hypothesis by showing increased levels of harman in the cerebellum of ET patients compared to healthy individuals [30]. It appears that the increased levels of this alkaloid may result from the excessive consumption of βCA-rich food (mainly heat-treated meat), the impaired ability to metabolize harman (a genetic cause), and/or increased endogenous harman production (genetic and environmental causes), possibly leading to the development of ET [8].

### 2.3. The Pathophysiology of ET

The pathophysiology of ET, as well as the etiology of the disease, has not been fully understood to date. The results of surgical, neurophysiological, and postmortem studies in ET patients, as well as studies in animal models, indicate that brain structures such as the inferior olive nuclei (ION), cerebellum, red nucleus, thalamus, and cerebral cortex (the cerebello–thalamo–cortical network) are involved in the pathomechanism of this disease [31]. Glutamatergic neurons arising from the ION form synapses on GABAergic PCs in the cerebellar cortex, and on glutamatergic neurons of the deep cerebellar nuclei (DCN) [31]. Furthermore, PCs control DCN activity either by the direct inhibition of the PC-DCN projection, or indirectly by the inhibition of GABAegic interneurons that form synapses with the glutamate projection neurons of the DCN [32,33]. Therefore, the signal coming from the cerebellum is the resultant activity of many types of cells in both the cerebellar cortex and nuclei. From the DCN, the nerve impulse travels further through the nuclei of the thalamus to the cerebral cortex, or directly to the red nucleus [31]. However, the exact brain mechanisms and structures involved in the generation of tremor or functional changes in the cerebello–thalamo–cortical network are not fully understood. Therefore, there are several different hypotheses regarding the mechanisms of tremor generation, the most popular of which are: (1) the neurodegenerative hypothesis; (2) the central oscillatory network hypothesis; and (3) the GABAergic hypothesis. Each of them are both accepted and criticized by researchers, and therefore there is still an open debate to determine the proper pathomechanism of ET [31,34].

#### 2.3.1. The Neurodegenerative Hypothesis

The progressive nature of the disease and the close relationship with age (ET mainly affects the elderly) are the main arguments in favor of the neurodegenerative basis of ET. Furthermore, it appears that ET may be associated with a greater likelihood of developing other neurodegenerative diseases, such as PD or Alzheimer’s disease [35]. Interestingly, it has been suggested that the mechanisms underlying ET and tremor in PD partially overlap. Moreover, symptoms of both diseases appear simultaneously quite often in up to 10% of cases [36]. The fundamental question regarding the neurodegenerative nature of the disease is whether there are characteristic histopathological changes in the brain. Postmortem studies have shown a wide variety of PC abnormalities, such as the swelling, thickening, and remodeling of the axons [37,38,39], regressive changes in the dendrites and a loss of spines [37,40], and the presence of heterotopic PCs (whose cell bodies are mislocalized in the molecular layer) [40,41,42]. Some studies reported significant PC degeneration [40,43,44,45], but there are also a few works that deny it [46]. The results of the research by Lin et al. [47] seem to be interesting as they showed that the synaptic connections between the ION climbing fibers and the cerebellar cortex PCs of patients with ET differed from those observed in healthy controls. Under normal conditions, climbing fibers form synapses mainly in the thicker and proximal dendrites of PCs, while in patients with ET, the reorganization of these connections is observed and climbing fibers begin to communicate with PCs through synapses located in the thinner and more distant dendrites, and, furthermore, these changes clearly correlate with the severity of tremor [47,48]. In addition to PC dysfunctions in ET, there are also disturbances in other neighboring nerve cells, e.g., the remodeling of basket cell axons [49,50] or an increase in Bergmann’s glial cells [51] and alterations in astroglia [52], which could be a compensatory response to restore cerebellar GABAergic tone and cerebellar cortical inhibitory efficacy [53]. However, reports from neuroimaging studies are ambiguous. According to the latest meta-analysis conducted by Holtbernd and Shah [54], there are as many studies that confirm (n = 14) as well as those that deny (n = 14) cerebellar atrophy during the course of the disease, so there is no clear evidence for cerebellar neurodegeneration. However, diffusion tensor imaging clearly shows microstructural changes in the cerebellum [54].

As for the mechanism of neuronal degeneration in the course of ET, it seems to be mainly related to excitotoxicity due to the persistent overactivity of the glutamatergic system. This is confirmed, among others, by elevated glutamate levels in the cerebrospinal fluid of patients with ET [55] or an increase in Glx (glutamate + glutamine) concentrations in the ventral intermediate nucleus (VIM) of the thalamus of ET patients, indicating a marked increase in thalamic glutamate transmission [56].

#### 2.3.2. The Central Oscillatory Network Hypothesis

Another hypothesis states that a central oscillatory network causes and drives tremor. This idea is based on the unique electrical properties of some central nervous system (CNS) neurons in which the hyperpolarization of the membranes causes them to oscillate (each cell independently of the others) at a given frequency. For a long time, the main goal in studying the pathomechanism of ET was to look for a single oscillator, a specific anatomical structure that causes tremor. The role of the tremor generator was initially assigned to the ION, the structure which is the starting point of the glutamate climbing fibers that form synapses on the cerebellar PC dendrites. ION neurons are characterized by a large number of electrical synapses containing connexin 36 (Cx36) that provides for the subthreshold network oscillations and for the synchronous and rhythmic activation of PCs by olivary climbing fibers [57].

The ‘olive theory’ of ET has been formulated primarily on the basis of studies in animal models of essential tremor, namely, the tremor induced by peripheral harmaline administration. Neurophysiological studies indicate that the ION is the primary site of harmaline influence in the CNS [58]. However, so far only a few studies have indicated functional abnormalities in the ION in patients with ET, including increased glucose metabolism [59], increased cerebral blood flow [60], and reduced regional homogeneity [61], which could indicate the hyperactivity of the examined brain structures. However, it should be noted that the functional abnormalities in these studies were not limited to the ION, and several other structures, including the cerebellum [60,61], motor cortex [61], and thalamus [59,61] were also abnormal in patients with ET.

Today, researchers are more inclined to adopt the theory that ET is generated not by one, but by many different oscillators, partially independent of each other or cooperating and driving each other. The brain structures belonging to the central oscillatory network include, in addition to the aforementioned ION, the cerebellum, thalamic motor nuclei, and the motor cortex [31]. Data that support such a hypothesis were provided, among others, by studies of coherence (frequency domain correlation) between signals recorded simultaneously from electrodes located on the surface of the skull (magnetoencephalography, MEG) and EMG (electromyography) signals from a selected muscle, which revealed a network of areas consisting of the contralateral primary motor cortex, premotor cortex, thalamus, ipsilateral cerebellum, and brainstem that are consistent with muscle activity during episodes of tremor [62]. These studies are in line with previous results from neuroimaging studies, where the increased activation of these structures was observed in the course of ET [63]. Studies of tremor recorded at the same time in several different muscles showed a high coherence between the activity of muscles within one limb and a lack of consistency when comparing the muscle activity of different limbs; therefore, it is unlikely that tremor is caused by only one oscillator [64]. Another argument against the theory of a single tremor generator is the fact that there is a transient coherence between oscillations in the motor cortex (measured by an electroencephalogram, EEG) and muscles (measured by an EMG), and the occurring breaks do not affect the intensity of peripheral tremor [65]. 

Computer modeling (dynamic causal modeling) based on fMRI and EMG results carried out by Buijink et al. [66] aimed at studying both the internal activity of the brain structures of the cerebello–thalamo–cortical pathway and the integrity of the entire system in patients with ET showed that tremor caused an increase in external activity (the activation of connections between the cerebellum and thalamus) and an increase in internal activity within each of these structures, while activity between the motor cortex and the cerebellum was weakened. These results suggest that connectivity along the cerebello–thalamic and cortico–cerebellar pathways is clearly altered in the course of ET [66].

The hypothesis of the central oscillatory network could explain previously inconsistent data, e.g., why ET disappears after cerebellar stroke in some patients [67], while in others, the disease develops after an extensive cerebral hemispherectomy where large portions of the cerebellum are removed [68]. It is also consistent with the finding that damage to various structures within the cerebello–thalamo–cortical pathway (e.g., thalamic nuclei) can inhibit the symptoms of ET [67], which is in contradiction to the theory of a single oscillator. 

#### 2.3.3. The GABAergic Hypothesis

Another important hypothesis postulates that disturbances in cerebellar GABAergic transmissions are at the root of ET. This hypothesis assumes four main stages of the development of the disease symptoms: (1) the degeneration of GABAergic PCs in the cerebellar cortex, which leads to (2) the reduced inhibition of the DCN, (3) which, in turn, causes their hyperactivity and, as a consequence, causes (4) the rhythmic activation of the thalamic nuclei and the increased activity of the thalamo–cortical pathway. This, in turn, results in the appearance of tremors in the periphery [69].

There are several arguments for the idea that ET is associated with the abnormal function of the GABA inhibitory neurotransmitter system. Firstly, drugs that improve GABAergic transmission, such as primidone, topiramate, gabapentin, and ethanol have been shown to reduce ET [31,69], as well as the GABA-A receptor agonist muscimol, which is injected directly into the VIM nucleus of the thalamus [70]. Furthermore, a slight decrease in GABA levels, with a concomitant increase in glutamate, in the cerebrospinal fluid of ET patients compared to controls was observed [55]. Postmortem studies using specific autoradiographic ligands for GABA receptors have shown that in patients with ET there is an approximate 22–35% decrease in the number of GABA-A and GABA-B receptors in the dentate nucleus of the cerebellum [71]. Furthermore, an inverse correlation has been shown between the GABA-B receptor binding and the duration of the disease, suggesting that with ET development, the number of these receptors gradually decreases [71]. In the brains of patients diagnosed with ET, the level of the GABAergic interneuron marker parvalbumin was also reduced, and this effect was observed only within the pons and locus coeruleus, but not in the cerebellum [72]. The neuroimaging studies using positron emission tomography (PET) with [^11^C]flumazenil, a ligand that specifically binds to the benzodiazepine site on the GABA-A receptor, yielded contrasting results. The authors of two independent studies observed the increased binding of [^11^C]flumazenil in the cerebellum, thalamus, and premotor cortex [73,74], suggesting an increase, rather than a decrease, in GABA-A receptors. Helmich et al. [31] suggested that in patients with ET, both the number of GABA-A receptors was reduced as well as their function was impaired or modified, hence resulting in changed affinity of [^11^C]flumazenil for these receptors. However, recent neuroimaging studies have reported no changes in GABA or Glx (glutamate + glutamine) levels in the dentate nucleus [75,76] or the thalamus [77] in patients with ET compared to controls, but there was a positive correlation of the GABA/Glx ratio with the severity of tremor [77].

It should be emphasized that the observed abnormalities in GABAergic transmission does not have a genetic basis, since to date no relationship has been found between the polymorphism of receptors and transporters for GABA and ET [31,69,78].

### 2.4. The Current Treatment of ET

#### 2.4.1. Pharmacological Therapy

Despite the increasing knowledge of the pathophysiology of ET, its treatment is still only symptomatic. Interestingly, all the drugs currently used in the treatment of ET were initially developed and approved for completely different disease entities, and their anti-tremor effect was discovered by accident. According to the criteria proposed by Hedera et al. [79], the substances used in the treatment of patients with ET can be divided into first-, second-, and third-line drugs. First-line therapies include propranolol (a nonselective beta-adrenergic antagonist) and primidone (an antiepileptic drug, a derivative of barbituric acid) (Table 1). To date, propranolol is the only substance approved by the Food and Drug Administration (FDA) in 1967 for the treatment of ET. It has been suggested that the mechanism of the anti-tremor effect of propranolol is based on blocking the peripheral non-cardiac β2 receptors located in the muscle spindles [80]. As a result of the fact that propranolol is a highly lipophilic substance and easily crosses the blood–brain barrier, its central effect cannot be excluded. Furthermore, a recent study using [^18^F]fluorodeoxyglucose PET showed a decrease in the regional glucose metabolism in the basal ganglia (BG) of patients with ET that responded to β-blockers, compared to those who did not respond to treatment [81]. Regarding the mechanism of the action of primidone in the context of ET, it is still unknown. Both primidone and its two active metabolites, phenylethylmalamide (PEMA) and phenobarbital, reduce tremor, but the effect of primidone is definitely stronger, and the administration of metabolites alone is not of therapeutic importance [82]. Both drugs are moderately effective. The therapeutic effects of propranolol and primidone are comparable, as both drugs reduce tremors by 50–70%. However, they differ in their response rate; with propranolol, improvements are seen in more than half of the patients with ET, while only 30–50% of patients respond to primidone. Both drugs are also characterized by a large number of side effects. Propranolol can induce hypotension, bradycardia, fatigue, erectile dysfunction, somnolence, and exercise dyspnea in 60% of patients, and 22–72% of patients treated with primidone exhibit sedation, fatigue, dizziness, ataxia, confusion, nausea, and flu-like symptoms [79]. 

The drugs that are likely to be effective in treating the symptoms of ET, that is, second-line drugs, include topiramate or some benzodiazepines (clonazepam and alprazolam) as well as other β-blockers, besides propranolol, such as atenolol and metoprolol. Among the listed substances, benzodiazepine drugs appear to be the most promising, since they are characterized by a good therapeutic effect (approximately a 50% tremor reduction) and a fairly high response rate to the drug (50–75%). However, benzodiazepines should be used with care due to their side effects and the risk of addiction [79]. In turn, topiramate was shown to reduce ET only in 29% of patients (16% in the placebo group) [85]. Third-line drugs, that is, drugs with a possible but not proven efficacy, include nimodipine (a dihydropyridine-sensitive calcium channel blocker) and clozapine (an atypical neuroleptic) [79]. According to the MDS Task Force on Tremor and the American Academy of Neurology, there is insufficient evidence to support or deny the use of clozapine in ET [84]. Several novel T-type calcium channel (Ca_v_3) blockers are currently being investigated, such as CX-8998 (Jazz Pharmaceutical, Dublin, Ireland) and PRAX-944 (Praxis Precision Medicines, Boston, MA, USA), which are at the stage of clinical trials [83]. In addition, a positive allosteric modulator of small conductance Ca^2+^-activated K^+^ channels (K_Ca_), CAD-1883 (Cadent Therapeutics, Cambridge, MA, USA) is in the phase II trial for ET [86]. The Sage Therapeutics company (Cambridge, MA, USA) has developed a series of allosteric modulators of GABA-A receptors, several of which have been evaluated for ET, e.g., brexanolone, Sage 217, and Sage 324. The latter was qualified for phase II clinical studies [83].

In general, the pharmacotherapy of ET in the elderly, who are mainly affected by this disorder, is extremely difficult for two main reasons: firstly, due to the long duration of the disease and the gradual worsening of symptoms, which requires dose escalation, and secondly, due to the frequent coexistence of respiratory or cardiovascular diseases in the elderly, which significantly limits the use of some drugs (mainly β-blockers).

An interesting therapeutic option for ET which borders on pharmacotherapy is the use of botulinum toxin type A (BTX-A), which is a protein derived from *Clostridium botulinum.* Its main mechanism of action is the cleavage of the presynaptic SNARE protein (SNAP-25) which thus inhibits the release of acetylcholine from the presynaptic vesicles. The typical duration of its effect is 3 to 4 months. The number of open-labeled studies, and a few controlled clinical trials, have shown a significant effect on hand tremor (upper limbs) associated with ET but also with PD tremor, dystonic tremor, and others, with a low incidence of side effects, mainly transient weakness. Additionally, several studies have shown good therapeutic effects on head and voice tremor in patients with ET, although there is a limited number of controlled data. The utility of BTX-A therapy for ET and PD tremor has been questioned based on the high incidence of finger and hand weaknesses after the treatment due to the injection method. However, this side effect has been overcome after the introduction of new methods, like the Yale protocol and the computerized kinematic tremor assessment, which produced significantly less weakness compared to previously reported methods [83,84,87].

#### 2.4.2. Neurosurgical Methods

Neurosurgical intervention may be an alternative to pharmacotherapy, which turns out to be ineffective in more than 30% of cases. The surgical treatment methods for ET can be divided into two groups: non-lesional (neuromodulation) and lesional (thalamotomy).

Neuromodulation methods include deep brain stimulation (DBS) and repeated transcranial magnetic stimulation (rTMS). Most frequently, the VIM is the target in the DBS method of the treatment of ET, and less often, the posterior subthalamic area (PSA) or the zona incerta (ZI) are also targeted [88]. Electrical neurostimulation can be performed unilaterally or bilaterally. In unilateral DBS, tremor reduction is seen on the opposite side of the body in relation to the hemisphere of the brain where the electrode is implanted [89]. According to the meta-analysis prepared by Elble et al. [88], it was estimated that in patients with ET who underwent DBS within the VIM or PSA, an improvement of approximately 73–96% was observed which lasted up to 12 months after the procedure, and after 1 year the effectiveness of DBS decreased slightly but still remained at a fairly high level, reducing tremors by about 70%. According to research by Baizabal-Carvallo et al. [90], the therapeutic effect of DBS may persist for up to 13 years. Despite its high efficiency, DBS, like any neurosurgical intervention, carries the risk of serious complications, including side effects related to the surgery itself (such as perioperative infection, pneumonia, intracerebral hemorrhage, and pulmonary embolism) and those related to electrical stimulation (such as paresthesia, muscle contraction, dysarthria, limb ataxia, gait disturbance, and disequilibrium) [88]. 

A novel neuromodulation technique, namely rTMS, is non-invasive as it does not require opening the skull and therefore does not have serious side effects. Functional changes in the CNS under the influence of rTMS depend on the frequency of stimulation; high-frequency stimulation (>1 Hz) leads to an increase in cortical neuron excitability, while low-frequency stimulation (<1 Hz) has the opposite effect [91]. The main disadvantage of this technique is that its tremor attenuating effect is short-term. Studies by Popa et al. [92] showed that the bilateral stimulation of the cerebellum by rTMS (1 Hz), repeated daily for 5 consecutive days, reduced tremor in patients with ET, and this effect lasted up to 3 months after the end of stimulation. 

Techniques that lead to the targeted lesioning of the motor thalamic nuclei, especially in the VIM area, are the oldest surgical methods for treating ET. Today, there are many different thalamotomy techniques, which can be invasive, such as the classic radiofrequency thalamotomy (RFT), as well as non-invasive, such as laser-induced thermal therapy (LITT), the gamma-ray thalamotomy, also known as the gamma knife radio-induced thalamotomy (GK), and the magnetic resonance-guided focused ultrasound thalamotomy (MRgFUS) [93]. According to the literature, RFT reduced tremor by at least 75% compared to baseline; however, this procedure can cause a number of serious side effects such as dysarthria, verbal or cognitive defects, weakness, confusion, somnolence, and paralysis [94]. It should be noted that the MRgFUS thalamotomy is the most recently approved treatment for ET and in studies of its effects 4 years after surgery, it was proven to reduce the hand tremor score by approximately 56%, the disability score by 63%, the postural score by 70%, and the action score by 63% compared to baseline. Furthermore, the authors stressed that there were no permanent adverse effects throughout the 4-year follow-up period [95]. What is more, Kim et al. [96] compared the success of treatment and treatment-related complications for the different surgical techniques—RFT, DBS, and MRgFUS. They showed that at 12 months postoperatively, treatment success for each procedure was observed in 70.6, 84.2, and 78.3% of the patients, respectively. Therefore, the effectiveness of the methods was comparable. In turn, the complication rates differed between treatment techniques and were significantly lower in the MRgFUS group, as only 4.4% of patients exhibited side effects 12 months after surgery compared to 11.8% for the RFT group and 21.1% for the DBS group [96], which makes this techniques extremely promising.

### 2.5. Animal Models of ET

#### 2.5.1. Harmaline-Induced Tremor

Harmaline tremor, which is a tremor induced pharmacologically by a single peripheral administration of harmaline, is the best known and the most common model of ET [97]. Harmaline is a substance of natural origin, belonging to the aforementioned group of βCAs, and is a tremorogenic compound that causes dose-dependent tremor in various animal species such as mice, rats, rabbits, cats, sheep, monkeys, and pigs [98,99,100]. The frequency of harmaline tremor is highly dependent on the size of the animal; for example, in monkeys it is 8–10 Hz, in rats it is 10–12 Hz, and in mice it is 11–14 Hz [101]. The tremor in the harmaline model appears in a few minutes after subcutaneous or intraperitoneal administration and lasts up to several hours [98]. Harmaline-induced tremor, similarly to ET, is an action tremor which is, more precisely, kinetic and postural [101], so it appears both while holding a selected position and while performing a voluntary movement. Furthermore, harmaline tremor is clearly intensified during movement. In mice and rats, tremor affected all four limbs, the tail, trunk, and head, including whiskers; thus, practically the entire body [98]. The exact mechanism by which harmaline induces tremor at the cellular level is not fully understood. Harmaline is a potent inhibitor of MAO-A, with low IC50 values (4–8 nM) [98]. The activity against MAO-B is negligible. Regarding receptor interactions, harmaline has an affinity for the 5-HT2A and 5-HT2C receptors (K_i_ = 7.8 and 9.4 µM, respectively) [102], but its contribution to tremor seems irrelevant, as harmaline action was not blocked by the 5-HT2A antagonist LY53857 [103]. Harmaline was also observed to displace [3H]flunitrazepam from the GABA-A receptor [104], but again, antagonists of these receptors (Ro15-1788, CGS8216) did not inhibit harmaline tremor [105]. Another study showed that harmaline also displaced [3H]MK801 from the NMDA receptor in rabbit ION fractions, suggesting that harmaline may induce tremor by acting as an inverse agonist at the MK-801 binding site on the NMDA receptor [106]. However, in this case depolarization should occur, while the hyperpolarization of the ION neurons was observed after harmaline [98]. At present, the most likely mechanism of tremor induction after harmaline appears to be the modulation of the T-type Ca^2+^ channel. Park et al. [107] proved that the selective knockdown of the *Cav3.1* gene in the ION efficiently suppressed harmaline-induced tremor in wild-type mice.

As mentioned above, ET is a disease that mainly affects the elderly, and as the patient gets older and the disease develops, the symptoms worsen. To model ET, acute harmaline administration is used, which is dictated by a very fast development of tolerance in the case of repeated administration. Studies by Wang and Fowler [108] indicated that on the second day of harmaline administration at different doses (4–16 mg/kg), no increase in tremor was observed in rats in the frequency range of 5–15 Hz, which is thought to be caused by both functional changes in the olivocerebellar pathway, resulting from damage to PCs in the cerebellar cortex, or a learned avoidance of movement by animals to prevent tremor [101]. Interestingly, a similar tolerance to repeated exposure to harmaline was observed in pigs, but not in mice [99,109]. However, it seems that harmaline tremor, similar to ET, develops only in adults, as evidenced by the results of neurodevelopmental studies showing that in rats and rabbits in the first week after birth, that is, in the period when the olivocerebellar system was still developing, harmaline did not cause tremor and its tremorogenic effect was observed only after the end of the formation of this system [100].

**Figure 1:**
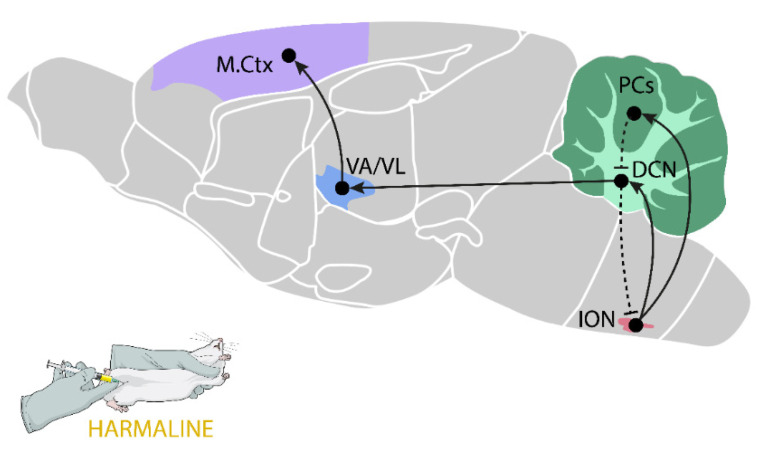
Neural circuits involved in the generation and spread of harmaline-induced tremor in rats. Description in the text. Solid lines: glutamatergic pathways, dashed lines: GABAergic pathways, DCN: deep cerebellar nuclei, ION: inferior olive nuclei, M.Ctx: motor cortex, PCs: Purkinje cells, VA/VL: ventroanterior/ventrolateral thalamic nuclei.

It is assumed that the primary site of harmaline action is the ION (Figure 1), especially the caudal medial and caudal dorsal accessory olive subnuclei (MAO, DAO) [110]. ION neurons have the ability to oscillate with a frequency in the range of 0.5–12 Hz, and the synchronization of discharges between individual ION neurons is possible due to the dendrito–dendritic electrical synapses (gap junctions) [111]. Harmaline that acts on the ION leads to the abnormal synchronous activation of glutamatergic climbing fibers that arise from the ION and form synapses in the PCs of the cerebellar cortex [101]. The activation of the climbing fibers induces complex spikes in PCs [112] and increases glutamate release in the cerebellar cortex [58,113]. The collaterals of the climbing fibers also go directly to the DCN, which sends excitatory glutamate projections to the ventral motor nuclei of the thalamus and then to the motor cortex [114,115,116]. Generally, the glutamatergic pathways are the main neural circuits responsible for tremor after harmaline, which is confirmed by harmaline-induced increased levels of extracellular glutamate in vivo in most of the above structures, that is, the cerebellar cortex [58,113], cerebellar nuclei [117], motor thalamus [118], and cerebral cortex [119]. In line with the above thesis, there are data indicating that some drugs, which decrease glutamatergic transmission, at the same time inhibit harmaline tremor. These are, among others, different antagonists of NMDA (2-APV, d-CCPene, MK- 801, PCP, and memantine) [120,121,122,123,124] and AMPA receptors (RPR117824 and NBQX) [123,125]. 

Several studies have proved the indisputable role of the ION in the generation of tremor after harmaline. In 1976, Simantov et al. [126] proved that selective damage to olivary climbing fibers by the administration of 3-acetylpyridine completely eliminated harmaline tremor. Furthermore, immunohistochemical studies indicated that harmaline caused an increase in *c-fos* immunoreactivity in the ION (15 min after peripheral administration), DCN (30 min), and the cerebellar cortex (1 h) [58,127], which showed an increase in neuronal activity in the examined brain structures. Furthermore, 3 h after harmaline injection, an increase in *c-fos* immunoreactivity was also observed in the striatum and the subthalamic nucleus [128]. In turn, in situ hybridization studies showed that harmaline increased the expression of *zif-268* mRNA in the ION, cerebellar cortex, ventroanterior/ventrolateral (VA/VL) nuclei of the thalamus, and the motor cortex, 1 h after administration [129]. 

In light of the research conducted so far, it seems that the DCN play a double role in modulating harmaline-induced tremor. In in vitro electrophysiological studies using a guinea pig section of the cerebellum and brainstem, harmaline administration was observed to lead to the activation of some DCN neurons, while other neurons within this structure were inhibited. This may suggest that harmaline stimulates both the activity of glutamatergic neurons emerging from DCN, leading to the generation of tremor, and the inhibitory GABAergic neurons in the DCN-ION pathway, modulating the rhythmicity and synchronization of climbing fiber oscillations [130]. 

In turn, the contribution of the thalamus to the induction of harmaline tremor is evidenced by the results of the study by Battista et al. [131], who observed that the lesion of the VL part of the thalamus attenuated harmaline-induced tremor in monkeys. Furthermore, Bekar et al. [132] showed that DBS with an electrode placed within the same area of the thalamus inhibited harmaline tremor in rats. 

Recently, Lee and Chang [133] measured single-unit neuronal activities in the resting state of the primary motor cortex during VL thalamic DBS in harmaline-induced tremor in rats and analyzed the neuronal activity patterns in the thalamo–cortical circuit. They reported that harmaline affected the neuronal firing rate in both the VL thalamic nuclei and primary motor cortex, and these patterns of activity were modulated by VL thalamic DBS, which proved the undisputable involvement of the motor cortex in harmaline-tremor generation. 

The above-described neuronal hyperactivity along the ION–cerebello–thalamo–cortical pathway led to the rhythmic stimulation of spinal neurons that formed synapses with muscle fibers (α-type neurons) and with muscle spindles (γ-type), and consequently leads to rhythmic muscle contractions observed as tremor [98,134].

Harmaline-induced tremor is an established model that is useful in the preclinical screening of potential ET therapies. Most drugs proven to attenuate harmaline tremor in an animal model also show a positive therapeutic effect in patients with ET. Concordance in the antitremor effect between the harmaline model and some patients with ET was demonstrated for propranolol, primidone, clonazepam, zonisamide, and gabapentin, among others. However, there is also a group of compounds that inhibit harmaline-induced tremor but do not affect ET (e.g., L-DOPA, lithium, and carbamazepine) or even worsen the symptoms of this disease (valproate) [98].

#### 2.5.2. Genetic Models

Among genetic models that exhibit ET-like symptoms, double knockout (KO) mice with a disruption of the α1 (α1−/−) subunit of the GABA-A receptor gene belong to more interesting studies [135]. The concept underlying this type of model is related to the GABAergic hypothesis of the pathomechanism of ET (see Section 2.3.3). About half of the total number of GABA-A receptors in the CNS contain α1 subunits, which are important both in the formation of the receptor complex itself and in the binding of benzodiazepines. In mice with a deletion of the α1 subunit, a decrease of approximately 50% in the density of GABA-A receptors in the brain was observed, as evidenced by the reduced binding of selective ligands ([^35^S]TBPS and [^3^H]Ro15-4513) in the cerebral cortex, thalamus, BG, hippocampus, brainstem, and cerebellum [136]. Behaviorally, GABA-A α1−/− KO mice are characterized by kinetic and postural tremor and impaired coordination. In this model, as in ET, tremor is reduced under the influence of propranolol, primidone, ethyl alcohol, and gabapentin [135]. Kralic et al. [135] also observed that MK-801 (a noncompetitive NMDA receptor antagonist) and CCPA (an adenosine A1 receptor agonist) inhibited tremor in GABA-A α1−/− KO mice, while compounds that enhance GABA-A receptor activity by increasing GABA affinity, such as diazepam and allopregnanolone, intensified tremor. This observation is somewhat inconsistent with clinical data which shows that diazepam, like other drugs in the benzodiazepine group, reduces the symptoms of ET in some patients [83,84].

Recently, research has been carried out on a new potential genetic model of ET, namely on *hotfoot17J* or Grid2^dupE3^ mice, which have a genetic mutation of the *Grid2* gene (a duplication of exon 3), leading to the mislocalization of the glutamate receptor delta 2 (GluRδ2), and accelerated protein degradation, mimicking the reduced expression of the GluRδ2 protein in the cerebellum of patients with ET [137]. The GluRδ2 receptor was found to be one of the key proteins controlling the territorial distribution of the climbing and parallel fiber synapses on the PC dendrites in the cerebellum [137]. In the absence or partial reduction of GluRδ2, the climbing fibers start to produce abnormal transverse branches extending mediolaterally to innervate the distal dendrites of neighboring and remote PCs, so that many PCs are wired by a single climbing fiber [138]. The described climbing fiber-to-PC synapse pathology is parallel to that observed in the postmortem ET cerebellum [137]. Moreover, recent studies have shown that mean GluRδ2 receptor expression is reduced by about 75% in patients with ET [137]. Grid2^dupE3^ mice developed a 20 Hz tremor that was suppressed by the systemic administration of primidone, propranolol, and ethanol [137,139].

On the one hand, the genetic models of ET appear promising due to symptoms similar to ET and its chronic character, as well as showing a good response to the first-line drugs used in the clinic. However, to date, there are no data on the effect of DBS or a thalamotomy in GABA-A α1−/− KO and Grid2^dupE3^ mice, which are highly effective in treating patients with ET.

## 3. The Dopaminergic System and Dopamine Receptors—The Role in ET

**Figure 2:**
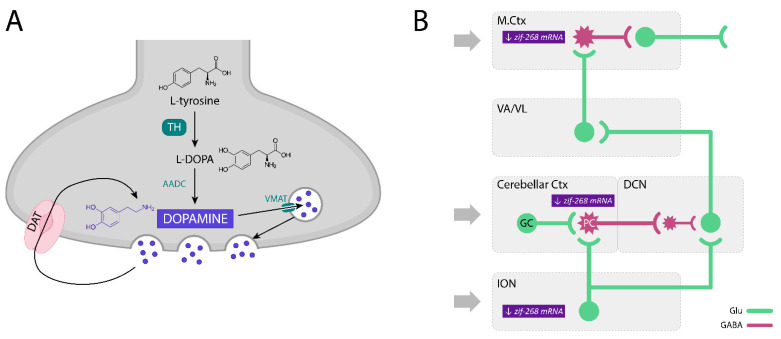
A scheme showing the process of dopamine synthesis within a dopaminergic neuron terminal (**A**), and brain structures potentially involved in the tremorolytic effect of pramipexole (**B**) (according to research [140]). AADC: aromatic L-amino acid decarboxylase (DOPA decarboxylase), DA: dopamine, DAT: dopamine transporter, DCN: deep cerebellar nuclei, GC: granular cell, ION: inferior olive nuclei, L-DOPA:L-3,4-dihydroxyphenylalanine (levodopa), M.Ctx: motor cortex, PC: Purkinje cell, TH: tyrosine hydroxylase, VA/VL: ventroanterior/ventrolateral thalamic nuclei, VMAT: vesicular monoamine transporter. Grey arrows indicate structures in which pramipexole inhibited *zif-268* mRNA expression increased by harmaline. For additional information, see the description in the text, Section 3.2.

Dopamine (DA) is the main catecholamine neurotransmitter, synthesized and secreted by the dopaminergic neurons of the CNS (see Figure 2A). These neurons form the dopaminergic systems, two of which are the best known: (1) the nigrostriatal system and (2) the mesocorticolimbic system, often divided into two distinct parts, the mesocortical part and the mesolimbic part.

Like other neurotransmitters, DA acts by activating specific receptors. Briefly, there are five subtypes of dopaminergic receptors, all metabotropic and coupled with the G protein, which are divided into two groups: (1) D1-like receptors (excitatory), linked to the Gαs/olf protein, which stimulates the activity of adenylate cyclase (AC) and cyclic adenosine-3′,5′-monophosphate (cAMP) production; and (2) D2-like (inhibitory) receptors, bound to the Gαi/o protein, which inhibits AC activity and cAMP production. The D1 family consists of D1 and D5 receptors, while the D2 family consists of D2, D3, and D4 receptors. All dopaminergic receptors are postsynaptic receptors, and D2 and D3 receptors may also be presynaptic [141,142].

### 3.1. The Participation of Dopamine and Its Receptors in the Modulation of ET and Harmaline-Induced Tremor

As already mentioned in this review, the cerebellum is one of the most important brain structures involved in both ET and the animal models of this disease, i.e., harmaline-induced tremor. The excitability of cerebellar neurons has been shown to depend mainly on glutamatergic transmission (see Section 2.5.1). However, there is evidence for the involvement of other neurotransmitters, including DA, especially in the context of the modulation of cerebellar function. It has been proven that all cerebellar layers receive a significant amount of dopaminergic afferents, of which the molecular layer is the most innervated [143]. The sources of cerebellar DA include dopaminergic neurons from the group A8 (red nucleus), A9 (substantia nigra pars compacta, SNc), and A10 (ventral tegmental area, VTA), as evidenced by studies by Kizer et al. [144], who observed an approximately 50% decrease in the DA level in the cerebellum as a result of bilateral lesions in these areas. It was also found that the cerebellar DA might act as a precursor of noradrenaline (NA), which is synthesized in the terminals originating from the locus coeruleus nuclei, but also as an independent neurotransmitter. This was suggested by Ikai et al. [145], who showed that the VTA sent bilateral projections both to the cerebellar cortex and to the DCN, while the VTA-DCN neuronal projections were not dopaminergic. Dopaminergic VTA neurons mainly reached the cells of the granular layer and the PC layer of the cerebellar cortex.

Moreover, although the levels of dopamine D1, D2, D4, and D5 receptors in the cerebellum are low, dopamine D3 receptors are relatively abundant in this structure, especially in PCs and their dendrites in the lobules 9–10 of the cerebellum [146,147], where they are involved in the regulation of locomotor activity [148,149,150]. Furthermore, the dendrites of GABAergic PCs in the cerebellar cortex express not only tyrosine hydroxylase (TH), the rate-limiting enzyme of catecholamine biosynthesis responsible for catalyzing the conversion of the amino acid L-tyrosine to L-3,4-dihydroxyphenylalanine, L-DOPA, a precursor of DA [151], but also the vesicular dopamine transporter, VMAT2, and the dopamine transporter, DAT, which may suggest their involvement in both GABA and DA transmission [151,152,153]. Therefore, dopamine D3 receptors may also serve as autoreceptors that inhibit DA release from the dendrites of PCs and thus regulate their activity [154]. 

The modulating effect of DA on cerebellar neuron activity was confirmed by the increase in the mRNA expression of the early response gene, *c-fos,* which was induced by the peripheral administration of the preferential D3 receptor agonist, 7-OH-DPAT [155]. Additionally, Heman et al. [156] showed that neurodegeneration of the nigrostriatal pathway induced a prolonged activation of PCs. The above effect may result directly from changes in dopaminergic transmission in the cerebellum [157] or be secondary to changes in other brain structures, e.g., structures of the BG, from where signals can be transmitted to the cerebellum via the multisynaptic neuronal pathways [158].

Dopamine D3 receptors, which appear to be of particular interest in the context of ET, are expressed, besides the cerebellum, in the ION, thalamus, and cortex. Thus, they are in all the brain structures important for ET and harmaline-induced tremor [159]. At present, however, little data is available on the role of dopaminergic transmission in ET, and only two postmortem studies are available. Rajput et al. [160] described a decrease in DA levels in the caudal region of the caudate nucleus, while another study by Shill et al. [72] reported similar levels of TH in the striatum of 23 ET patients, compared to 37 control individuals. 

Most of the functional studies on the dopaminergic system performed in patients with ET did not show alterations in tracer uptake (unlike in PD) compared to healthy controls. Several studies using ligands for DAT markers of neuronal lesions or postsynaptic D2 receptors, including the SPECT or PET scans, found only a mild striatal dopaminergic deficit, or an absence of any significant alterations in these markers in patients with ET. For detailed information about studies on the catecholaminergic system in ET, see Table 2. 

On the other hand, the results of the clinical trials conducted in a small group of ET patients indicated the potentially important role of the D3 receptor in ET modulation. Herceg et al. [174] observed a marked and significant attenuation of tremor (approx. by 52%) in patients after administration of pramipexole, the preferential agonist of these receptors.

### 3.2. Involvement of Dopamine D3 Receptors in the Harmaline-Induced Tremor

In addition to stimulating the olivo-cerebellar climbing fibers, harmaline is also an inhibitor of monoamine oxidase-A (MAO-A) [175], an enzyme involved in DA metabolism. The results of a study by Ossowska et al. [176] indicated that the non-selective dopamine receptor agonist, apomorphine, increased harmaline tremor in rats. Together with the inhibition of MAO-A function, this could suggest that increased dopaminergic transmission is important in the mechanism of tremor generation, including in this ET model. On the other hand, Paterson et al. [123], in a study carried out in mice, observed that apomorphine and quinpirole (a D2/D3 receptor agonist) inhibited tremor after harmaline, while SKF 82,958 (a D1 receptor agonist) and raclopride (a D2 receptor antagonist) did not show any significant effect. These authors also showed that injection of GBR 12902, a DAT inhibitor, attenuated harmaline tremor, but only at one of the doses used, while after the administration of both lower and higher doses no similar effect was observed [123]. 

Contradictory data are also provided by studies examining the influence of a midbrain dopaminergic neuron lesion on the intensity of tremor after harmaline. The 6-OHDA lesion in the A9 field (SNc) and A8 (red nucleus) increased tremor caused by a low dose of harmaline [157] or inhibited tremor after a high dose of this compound [113]. 

Recently, Kosmowska et al. [177] showed that pramipexole, a known anti-parkinsonian drug that inhibits resting tremors in patients with this disease [178], at the lowest dose used, reduced harmaline-induced tremor in rats measured using a force plate actimeter (FPA). Like propranolol, a well-known drug of first choice in the treatment of ET, pramipexole significantly reduced the power of tremor at a frequency of 9–15 Hz (AP2), i.e., in the range characteristic of harmaline tremor [177]. However, at higher doses, this drug did not affect tremor in this rat model of ET [177]. Since Herceg et al. [174] found that pramipexole reduced tremor in a small number of patients with ET, these studies by Kosmowska et al. [177] seem to confirm the potential usefulness of pramipexole in the treatment of ET and the suggest continuation of clinical trials using this drug.

Moreover, Kosmowska et al. [177] showed that another high-affinity agonist of dopamine D3 receptors, 7-OH-DPAT, also reversed the harmaline-increased AP2 parameter, so, like pramipexole, it was effective in reducing harmaline tremor. Therefore, the involvement of dopamine D3 receptors in harmaline-induced tremor seemed highly probable. To search for a putative mechanism, the authors used different antagonists of the D3 and D2 receptors, such as SB-277011-A and SR-21502 (D3 antagonists), haloperidol (a non-selective D2 antagonist), and amisulpride (a D2 and D3 autoreceptor antagonist in vivo). None of the dopamine receptor antagonists examined by the authors influenced the tremorolytic effects of pramipexole or 7-OH-DPAT [177]. Therefore, Kosmowska et al. [177] concluded that the tremorolytic effect of pramipexole may suggest its beneficial effect in ET patients, however, the mechanisms underlying its action are unclear and need further examination [178]. 

In the following study, Kosmowska et al. [140] searched for brain structures involved in the tremorolytic effect of pramipexole and determined the expression of the early activity-dependent gene, *zif-268* mRNA, by in situ hybridization. They found that harmaline increased both the tremor and expression of *zif-268* mRNA in the IO, cerebellar cortex, ventral nuclei of the thalamus, and motor cortex [140]. Pramipexole reversed the harmaline-induced tremor and there was an increase in *zif-268* mRNA expression in all the structures examined except for the ventral thalamic nuclei [140]. Moreover, the tremor intensity was positively correlated with the expression of this early gene in all the structures mentioned above, suggesting that the tremorolytic effect of pramipexole is related to the modulation of the enhanced harmaline-induced neuronal activity in the well-known structures of the tremor network, including the ION, cerebellar cortex, and motor cortex [140] (Figure 2B).

In short, the preferential agonists of the dopamine D3/D2 receptors, pramipexole and 7-OH-DPAT, attenuated the harmaline-induced tremor, and this effect was not reversed by any of the antagonists of these receptors used by Kosmowska et al. [177]. Pramipexole also reduced the level of harmaline-increased expression of *zif-268* mRNA in the ION, cerebellar cortex, and motor cortex. These results obtained by the authors [140,177] confirmed the beneficial effects of pramipexole, a registered anti-parkinsonian drug, in the harmaline model of ET and its potential role in ET therapy. In addition, the attenuation of the harmaline-induced activity of the ION, cerebellar cortex, and motor cortex may be important in the tremorolytic effect of pramipexole, although the exact neuronal mechanisms underlying the drug action are unfortunately still unclear.

## 4. Adenosine and Its Receptors—The Role in ET

### 4.1. Adenosine Metabolism

Adenosine, an endogenous purine nucleoside present in all mammalian tissues, modulates a variety of important synaptic processes and regulates the functions of several neurotransmitters in the CNS. Adenosine is considered to be a neuromodulator rather than a neurotransmitter and it affects neural activity through multiple mechanisms: presynaptically, by controlling neurotransmitter release; postsynaptically, by hyperpolarizing or depolarizing neurons; and nonsynaptically, mainly via regulatory effects on glial cells [179,180,181,182]. 

**Figure 3:**
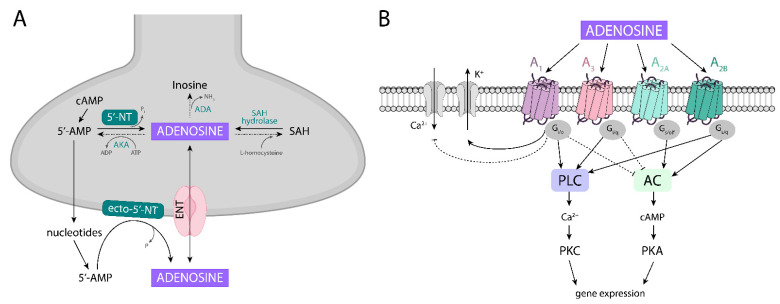
The synthesis and degradation of adenosine in the CNS (**A**), and adenosine A1, A2A, A2B, and A3 receptors, their activation and intracellular signal transduction pathways (**B**). Description in the text. A1, A2A, A2B, and A3: adenosine receptors, AC: adenylate cyclase, ADA: adenosine deaminase, ADP: adenosine 5′-diphosphate, AKA: adenosine kinase, ATP: adenosine 5′-triphosphate, cAMP: cyclic adenosine-3′,5′-monophosphate, ecto-5′-NT: ecto-5′-nucleotidase, ENT: nucleoside transporter, 5′-AMP: adenosine 5′-monophosphate, 5′-NT: 5′-nucleotidase, SAH: S-adenosylhomocysteine, PKA: protein kinase A, PKC: protein kinase C, PLC: phospholipase C (based on research [183,184,185]).

It is well known that adenosine may be formed in the CNS either intracellularly, and then transported by nucleotide transporters to the synapse, or extracellularly, from nucleotides released into the synaptic cleft (Figure 3A). Thus, the formation of adenosine is dependent on the availability of oxygen and energetic compounds, as well as on the rate of synthesis and the degradation of ATP released from both neuronal and glial cells. Moreover, adenosine can be directly released by nucleoside transporters from astrocytes when its intracellular level is augmented in response to a variety of physiological and pathological stimuli (e.g., increased cellular activity, hypoxia, hypoglycemia, and ischemia). 

In the cell, adenosine may be formed by the hydrolysis of 5′-AMP (adenosine-5′-monophosphate) catalyzed by the enzyme 5′-nucleotidase (5′-NT) which belongs to the family of enzymes called ecto-nucleotidase [185,186]. This pathway of adenosine formation through cytosolic ATP catabolism seems to represent a very sensitive signal of increased metabolic rates or metabolic stress [185]. Further, the hydrolysis of S-adenosylhomocysteine (SAH) by SAH hydrolase, an enzyme present in brain areas such as the neocortex, hippocampus, and cerebellum, may be an additional source of intracellular adenosine [185].

On the other hand, in the extracellular compartment, adenosine is formed either from synaptic ATP released after degradation to AMP, or from released cAMP after its conversion by ecto-phosphodiesterase to 5′AMP, and further to adenosine. Another possible way is that cAMP can be transformed inside the cell into 5′-AMP, which is then released into the synapse, becoming a source of adenosine. Outside the cell, adenosine is synthesized with the participation of ecto-5′-nuclotidase (ecto-5′-NT), present both in the nerve and glial cells [185,186].

In the extracellular space, adenosine levels are kept in equilibrium by specialized bi-directional transporters. There are two types of nucleoside transporters: (1) equilibrative nucleoside transporters (ENT, sodium-independent), which are dominant in the CNS and are responsible for transporting adenosine in and out of the cell; and (2) concentrative nucleoside transporters (CNT, sodium-dependent) [182,185,186,187]. 

The process of extracellular adenosine formation is very fast, and occurs within seconds [188]. Outside the cell, under physiological conditions, adenosine is present in a relatively low concentration (nM) which is sufficient to stimulate both the higher affinity A1 and A2A receptors. Under hypoxic/ischemic conditions and seizures, adenosine concentrations rise markedly to µM concentrations that can stimulate both the lower affinity A3 and A2B receptors [188,189]. It seems that in vivo, a large part of the adenosine present in the synapse, under basal conditions, comes from the extracellular metabolism of nucleotides [185,190].

The inactivation of extracellular adenosine following the uptake of the nucleoside back into the cell by the aforementioned transporters occurs by: (1) the phosphorylation of AMP by adenosine kinase (AKA); (2) the deamination of adenosine to inosine in a reaction catalyzed by adenosine deaminase (ADA); and (3) a reversible reaction catalyzed by SAH hydrolase leading to the synthesis of SAH from adenosine and L-homocysteine [184,185,190].

### 4.2. Adenosine Receptors, Their Localization, and Homomeric and Heteromeric Complexes

Adenosine released into the synaptic space acts through its receptors, which belong to the family of G protein-coupled receptors: A1, A2A, A2B, and A3 (Figure 3B). The A1 and A3 receptors preferentially couple to Gα_i/o_ proteins, and their stimulation leads to: (1) the inhibition of adenylate cyclase (AC), the reduction of cAMP production and thus the inhibition of protein kinase A (PKA) activity; and (2) the stimulation of phospholipase C (PLC), which regulates the activity of protein kinase C (PKC) and the internal concentration of Ca^2+^ ions. On the other hand, the activation of the A2A and A2B receptors (which bind to Gα_s/olf_ proteins) leads to the activation of AC and the increased release of cAMP. Additionally, an activated A2B receptor stimulates phospholipase C (PLC) activity. Regarding A1 receptors, apart from their effect on AC, their action may also involve other signal transduction mechanisms, e.g., the inhibition of voltage-dependent Ca^2+^ channels and the stimulation of K^+^ channels [182,184,191].

The most abundant adenosine receptors present in many regions of the CNS both presynaptically and postsynaptically are the adenosine A1 receptors. The highest expression of A1 receptors has been found in the cerebral cortex, striatum, thalamus, cerebellum, hippocampus, and spinal cord [181,192,193,194,195]. Moreover, their mRNA is also present in the structures of the BG, including the striatum, globus pallidus, and subthalamic nucleus [196]. These receptors are also present on glial cells (astrocytes, oligodendrocytes, and microglia) [180,197,198,199]. In the striatum, adenosine A1 receptors are present on both direct and indirect GABAergic efferent neurons as well as on cholinergic interneurons [200,201,202]. Furthermore, presynaptic A1 receptors are present on glutamatergic cortico-striatal and dopaminergic nigro-striatal afferents as well as on nerve terminals in the globus pallidus, substantia nigra, and hippocampus, where they modulate the release of many neurotransmitters, such as glutamate, acetylcholine, serotonin, and GABA [195,203,204]. These receptors are also present at high levels in the periphery (heart atria, kidneys, adipose tissue, and pancreas) and in several immune cells [182,191,205]. Such a broad distribution of A1 receptors reflects the involvement of these receptors in the regulation of many physiological functions, including, in addition to the release of neurotransmitters, effects on neuronal excitability, the control of the sleep/wakefulness processes, and many others, such as anticonvulsant and analgesic effects. 

In contrast to the widespread distribution of A1 receptors in the CNS, the A2A receptors are mainly localized in the striatum, nucleus accumbens, and olfactory tubercle. However, these receptors have also been found, albeit at lower levels of expression, in several other brain areas, such as the hippocampus, cerebral cortex, extended amygdala, thalamic nuclei, and substantia nigra [196,206,207,208,209,210,211,212]. It is noteworthy that A2A receptors are also present on glial cells [180,211,213,214,215].

In the striatum, A2A receptors are homogeneously distributed and are mainly localized postsynaptically on the GABAergic medium-sized spiny neurons of the indirect pathway projecting to the external segment of the globus pallidus, together with dopamine D2 receptors and enkephalin [207,210,211,216,217,218,219]. The A2A receptors in the striatum are also located presynaptically on glutamatergic terminals [211,220,221], where they heteromerize with A1 receptors and regulate glutamate release [220,222]. Such a co-expression of adenosine A2A and A1 receptor mRNAs was also found on the glutamatergic nerve terminals in the hippocampus [207], where these receptors may control glutamate release. Furthermore, A2A receptors located on striatal cholinergic nerve terminals modulate acetylcholine release [223,224,225]. 

Adenosine A2B receptors are present mainly in peripheral organs, like the bowel, bladder, lung, and vas deferens, but can also be found in the spinal cord and brain [226,227,228]. In the CNS, A2B receptors are present on the neurons of the hippocampus, hypothalamus, thalamus, and also in the striatum. Low levels of these receptors are also expressed on glial cells [180,226,227,228,229].

Regarding the distribution of A3 receptors in the brain, they are known to be present at a relatively low level on neurons, astrocytes, and microglia in the hippocampus, as well as the cerebral cortex, cerebellum, thalamus, and striatum [180,191,196,230,231,232,233]. These receptors are widely distributed in peripheral organs, mainly in the testis and lung, and in enteric neurons [196,234,235].

Both the adenosine A1 and A2A receptors are capable of combining to create receptor complexes in the form of homomers or heteromers by the interaction with receptors of the same type (A1-A1 and A2A-A2A) or of a different type [236,237,238]. Importantly, the properties of oligomers can differ significantly from those of the monomeric receptors because the functional nature of the receptor is largely dependent on its tertiary or quaternary structure. Therefore, receptor complexes are often characterized by different abilities to bind specific ligands. Adenosine A1 receptors have been shown to form heterodimers with the adenosine A2A [239], dopamine D1 [240], mGluR1 metabotropic glutamate [241], and purine P2Y1 receptors [242]. On the other hand, adenosine A2A receptors, besides adenosine A1 receptors, may also form heterooligomers with the dopamine D2 and D3 receptors [243,244], the mGluR5 metabotropic glutamate receptors [245], and the CB1 cannabinoid receptors [246]. 

### 4.3. The Involvement of Adenosine and Its Receptors in Tremor Modulation 

Among the adenosine receptors, A1 and A2A with a high nM affinity for adenosine appear to be the most important ones. Adenosine A1 receptors, as already mentioned above, are common in the brain and in the periphery, with the highest concentration in the cerebral cortex, thalamus, and cerebellum, and with slightly lower concentrations in the ION and BG nuclei [200], where their main function is neuromodulation, i.e., the regulation of neurotransmitter release. It has been proven that the stimulation of presynaptic A1 receptors leads to a reduction in the release of the main excitatory neurotransmitter glutamate, but also a reduction in acetylcholine, serotonin, dopamine, and noradrenaline, and in some brain areas, it may also inhibit the release of GABA [203,247]. A1 receptor-mediated actions are particularly important under non-physiological conditions (hypoxia and ischemia) when there is an excessive release of, e.g., glutamate, the increased and prolonged activity of glutamate receptors (both ionotropic and metabotropic) and an uncontrolled inflow of Ca^2+^ ions to cells. This increased inflow activates numerous enzymes, including phospholipases, endonucleases, and proteases such as kalpain, which damage cellular structures, the cytoskeleton, cell membranes, and DNA. Consequently, this leads to excitotoxic neuronal death [248]. Therefore, the reduction of glutamate release by adenosine A1 receptor stimulation (by adenosine or exogenous agonists) may possess neuroprotective effects [182,249,250]. On the other hand, it is known that the activation of presynaptic adenosine A2A receptors causes the opposite effect, which thus enhances the release of many neurotransmitters (glutamate, acetylcholine, serotonin, GABA, and noradrenaline). Hence, in this case, neuroprotective effects can be exhibited by A2A receptor antagonists [182,203,247,249,250]. In addition, the blockade of A2A receptors was shown to inhibit parkinsonian-like tremor, evaluated in the animal model through tremulous jaw movements (TJMs) [251,252].

At present, little is known about the participation of adenosine and its receptors in the modulation of ET. However, the adenosine A1 receptors seem to be especially interesting as a potential therapeutic target for tremor treatment, since they are present in all brain structures important for harmaline tremor. The first suggestion about the putative role of adenosine in tremor came from a single study by Sandyk [253] who found that in a patient with ET and asthma, acute treatment with aminophylline (a compound consisting of the bronchodilator theophylline and ethylenediamine in a 2:1 ratio), which is less potent than theophylline, alleviated both the respiratory distress and the tremor. However, studies by Buss et al. [254] on 10 ET patients who received an acute i.v. infusion of aminophylline did not confirm the antitremor effect of xanthine, and even a significant increase in finger tremor in those patients was found. Theophylline, the non-selective adenosine receptor antagonist which was supposed to worsen the tremor, was then tested in a small clinical trial of patients with ET and PD by Mally [255], who found a significant alleviation of tremor over a period of 2–4 weeks. In turn, Mally and Stone [256,257,258] conducted a small study on ET patients who were given theophylline in low doses chronically for 4 weeks and observed that this compound attenuated the tremor to a degree comparable to propranolol, the first-choice drug in ET. The beneficial effect of theophylline developed more slowly than that of propranolol, which was suggested by the authors to result from the chronic theophylline administration and the up-regulation of adenosine receptors with a resulting progressive decrease in neurotransmitter release. Moreover, in contrast to propranolol, no adverse effects of this drug were seen in ET patients. Additionally, studies on mice showed that the chronic effect of theophylline on the up-regulation of GABA receptor function was comparable to that of propranolol; therefore, it was suggested that the increased sensitivity to GABA after both treatments might reflect its role in reducing ET [258].

Another study carried out by Louis et al. [259] was based on the evaluation of the daily consumption of various substances, including caffeine, in patients with diagnosed ET. Caffeine, a non-selective antagonist of adenosine receptors, and a methylxanthine derivative, is metabolized to theophylline, which, in low doses, has been shown to decrease the severity of ET [257,258]. Moreover, lower caffeine consumption was associated with an increased risk of other movement disorders, especially PD [260]. Based on questionnaires filled out by patients, the researchers concluded that ET patients consumed much less caffeine compared to the control group, and consumption was not correlated with the severity or duration of the tremor. Therefore, the authors suggested that lower caffeine consumption in ET patients might not exclusively be a response to tremor, and more prospective studies are needed to test the hypothesis that a reduced caffeine intake may be a risk factor for developing ET, as it is for PD. Unfortunately, such hypothesis has not been confirmed in another independent study by Prakash et al. [261] on 179 subjects, including 79 ET patients and 100 controls matched for age, gender and ethnicity, where no significant correlation between caffeine intake and disease duration or total tremor score in ET patients was found.

Most of the existing knowledge on the potential role of adenosine comes mainly from studies in the animal model of ET, in which tremor is induced by harmaline. Al Deeb et al. [262] observed that caffeine given acutely 60 min after harmaline increased the intensity and amplitude of tremor and also prolonged the time of harmaline action in rats. These results, according to those authors, suggested that acute caffeine treatment in high doses of 50–100–150 mg/kg potentiated harmaline-induced tremor in rats. Since the GABAergic pathway was suggested to play an important role in ET in humans and in harmaline-induced tremor in rats, and adenosine modulates the release of GABA neurotransmission in various regions of the brain, according to Al Deeb et al. [262] the blockade of adenosine receptors by caffeine might interfere with the release of GABA, leading to exacerbation of the tremorogenic effect of harmaline. Moreover, since caffeine may increase the intracellular availability of calcium as a result of reduced uptake [263] or enhanced release [264], it may exacerbate the tremorogenic activity of harmaline by enhancing the availability of calcium. However, further studies are needed to identify the mechanism of caffeine action on harmaline-induced tremor. On the basis of all the results with methylxanthines, the MDS Task Force on Tremor considered that there is lack of sufficient evidence to support or refuse the use of theophylline in ET [265].

It has already been mentioned (see Section 2.4.2) that a thalamotomy and DBS are effective neurosurgical treatments for ET. What is interesting is that during DBS, the implantation of the electrode in thalamic nuclei can reduce tremor itself, before the pulse generation is activated. This microthalamotomy effect (the relief of tremor before neurostimulation) has been observed in 53% of patients undergoing DBS [266]. In turn, Bekar et al. [132], in studies on mice, showed that DBS within the VL thalamic nuclei (the human equivalent of the VIM nucleus where DBS is effective in alleviating both PD tremor and ET in humans) intensified the ATP release and, consequently, increased the synthesis of endogenous adenosine. Recently, Chang et al. [267] performed experiments with in vivo real-time wireless fast-scan cyclic voltammetry to quantify extracellular neurotransmitter concentrations in the brain of ET patients during DBS, and found that the microthalamotomy effect is accompanied by local neurochemical changes, including adenosine release.

Beckar et al. [132] also showed that the administration of exogenous adenosine or an adenosine A1 receptor agonist (CCPA) directly into the VL nucleus of the thalamus inhibited tremor caused by harmaline, while the antagonist of these receptors (DPCPX) intensified tremor. Moreover, they observed that both the pharmacological and genetic inactivations of adenosine A1 receptors caused involuntary movements and seizures at stimulation intensities below the therapeutic level. These results support the clinical data that caffeine can trigger or exacerbate ET [259]. Therefore, it seems that adenosine A1 receptors are of great importance for the therapeutic effect of DBS in the harmaline model of ET. In addition, in vitro studies by Kessler et al. [268] on the organotypical culture of the PCs of the cerebellar cortex indicated that the stimulation of adenosine A1 receptors, caused by the agonist R-PIA, inhibited the spontaneous discharges of these neurons. 

### 4.4. Adenosine A1 Receptors and Their Role in Harmaline-Induced Tremor

Recent results by Kosmowska et al. [118,129] confirmed the involvement of adenosine A1 receptors in the modulation of harmaline tremor in rats. The authors showed that the direct stimulation of A1 receptors with the brain-penetrant, potent, and selective agonist 5′Cl5′d-(±)-ENBA, which was given systemically, strongly, and dose-dependently, reduced harmaline-induced tremor measured automatically in FPA cages as the averaged power in the frequency band of 9–15 Hz (AP2 parameter) and tremor index (TI), which is a difference in power between parameters AP2 and AP1 (the averaged power in the frequency band of 0–8 Hz). Moreover, the tremorolytic effect of 5′Cl5′d-(±)-ENBA was reversed by a selective antagonist of the adenosine A1 receptors, DPCPX. However, DPCPX administered alone did not influence harmaline-induced tremor, indicating the lack of a modulating effect of endogenous adenosine on tremor. 

Harmaline has already been shown to induce the expression of the neuronal responsiveness markers such as early genes *c-fos* [58,127,128] and *zif-268* mRNA [129] in structures known to be involved in ET and harmaline tremor, such as the ION and cerebellar cortex. Moreover, Kosmowska et al. [118,129] observed an increase in the expression of *zif-268* mRNA in other structures such as the VA/VL thalamic nuclei and motor cortex, and all these effects were reversed by the stimulation of adenosine A1 receptors. However, the blockade of A1 receptors with DPCPX reduced this 5′Cl5′d-(±)-ENBA effect only in the motor cortex [129], a structure known to be a part of the oscillatory network active in ET. This suggests the involvement of cortical adenosine A1 receptors in the tremorolytic effect of this agonist, and a potential therapeutic target for ET treatment. 

It has been already mentioned that adenosine via A1 receptors reduces the excitatory synaptic transmission, glutamate release, and spontaneous neuronal activity in different brain structures. Such a mechanism may be responsible for the A1 receptor-induced inhibition of harmaline tremor, especially because the crucial role of the glutamatergic system in this tremor has already been suggested (for more information, see Section 2.5.1). In fact, the synchronous activation of glutamatergic olivo-cerebellar climbing fibers and an increase in glutamate release in the cerebellum [58,113], which triggers complex spikes in the PCs and oscillations in the DCN, are generally accepted to be the primary cause of tremor induced by harmaline. 

**Figure 4:**
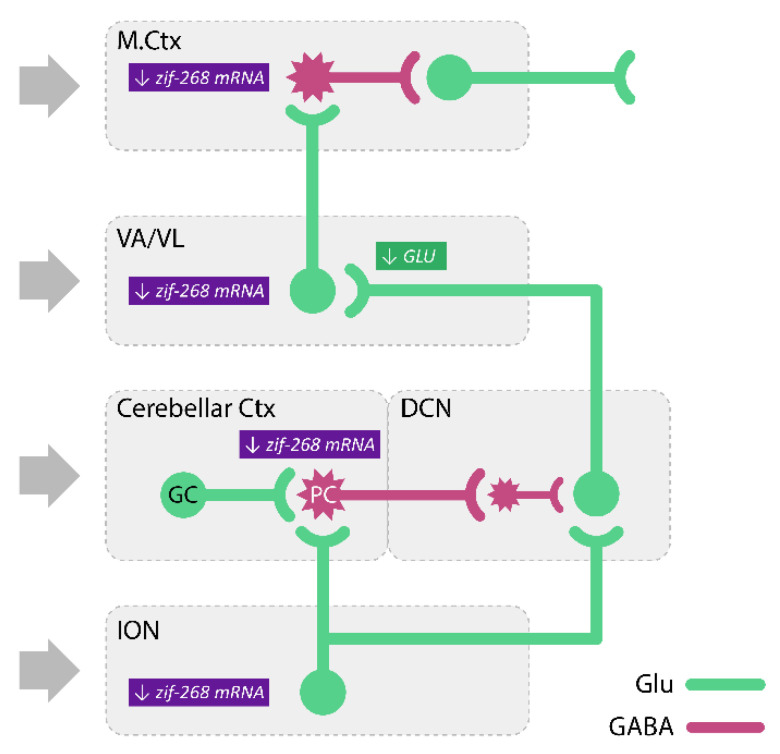
Scheme presenting brain structures involved in the effect of adenosine A1 agonist, 5′Cl5′d-(±)-ENBA, on the harmaline-increased *zif-268* mRNA expression as well as on the harmaline-enhanced glutamate release in the VA/VL nuclei of the thalamus. Grey arrows indicate areas of the brain potentially involved in the tremorolytic effect of 5′Cl5′d-(±)-ENBA, where effects on general neuronal activity and glutamate transmission have been observed (based on research [118,129]). DCN: deep cerebellar nuclei, GC: granular cell, ION: inferior olive nuclei, M.Ctx: motor cortex, PC: Purkinje cell, VA/VL: ventroanterior/ventrolateral thalamic nuclei. For additional information, see Section 4.4.

Subsequent studies by Kosmowska et al. [118] focused on the evaluation of the role of the glutamatergic system in the mechanism of the tremorolytic action of selective adenosine A1 receptors agonist in the harmaline model of ET. The authors demonstrated for the first time that harmaline increased glutamate release, measured by microdialysis in vivo in the motor nuclei of the thalamus (VA/VL) that partly corresponds to the VIM nucleus in humans. This effect of harmaline complemented current knowledge and confirmed that harmaline enhances glutamatergic transmission via the pathways connecting the ION, cerebellum, motor thalamic nuclei, and motor cortex (see Figure 1) [58,113,117,119] and it is in line with clinical data, which showed an increased concentration of Glx (glutamine + glutamate) in the VIM thalamic nucleus of ET patients in vivo, which was correlated with tremor intensity [56]. What is interesting is this effect of harmaline on glutamate release was decreased by the stimulation of adenosine A1 receptors with 5′Cl5′d-(±)-ENBA [118], which may suggest that the mechanism of the tremorolytic effect of the A1 receptor agonist is related to the weakening of glutamatergic transmission in the motor thalamus. However, since A1 receptors are quite common in the entire brain and occur in all brain structures involved in harmaline-induced tremor, the suppression of glutamatergic transmission at all levels of the tremor network could probably be important for the tremorolytic effect of the adenosine A1 agonist given systemically (Figure 4). 

All these findings suggest that adenosine A1 receptors might be a potential therapeutic target for the treatment of ET. Unfortunately, it is also known that stimulation of these receptors causes several undesirable effects such as sedation, hypothermia, and cardiovascular depression (reduced blood pressure and negative inotropic/dromotropic and chronotropic properties) in humans and animals [182,205], which can be extremely disadvantageous. Therefore, the clinical use of adenosine A1 agonists for ET treatment may be limited.

## 5. Conclusions and Perspectives

ET is one of the most common movement disorders characterized by severe tremor mainly in the upper extremities that can make the simplest of daily activities difficult for patients. Unfortunately, current pharmacotherapy is ineffective in more than 30% of patients. In more severe cases, DBS can be used, but it is associated with the risk of serious perioperative complications. Therefore, there is an urgent need to look for new therapies, which is certainly not facilitated by the fact that the pathomechanism of ET is not yet fully understood. One of the most established animal models of ET is based on the pharmacological induction of tremor by the peripheral administration of harmaline. The neuronal circuits responsible for the generation of tremor in this model mainly involve the glutamatergic pathways that connect the ION, cerebellum, ventral thalamus, and motor cortex. However, according to the research results presented here, it seems that other neurotransmitters may also be involved in the modulation of this tremor. In this review, we summarized the current knowledge on the influence of dopamine and adenosine, and the stimulation or blockage of their receptors, on tremor. Regarding the dopaminergic system, the stimulation of dopamine D3/D2 receptors using the preferential D3 receptor agonist pramipexole has been shown to attenuate harmaline-induced tremor in rats, as well as reducing tremor in a small group of patients with ET. Unfortunately, the putative mechanism of the therapeutic action of pramipexole in this kind of tremor is still unknown, but it has great therapeutic potential in ET, especially because it is already a registered anti-parkinsonian drug. 

The results of studies with non-selective adenosine receptor antagonists, such as theophylline and caffeine, provide conflicting data. Therefore, the MDS Task Force on Tremor concluded that there is a lack of sufficient evidence to support or refuse the use of theophylline in ET. On the other hand, there is also strong evidence suggesting that adenosine A1 receptors might be a potential therapeutic target for the treatment of ET, as a selective agonist of these receptors significantly reduced harmaline-induced tremor. The only problem with these types of compounds is that they could cause several side effects which are related to the presence of A1 receptors in the periphery. Therefore, so far, the clinical use of adenosine A1 agonists appears to be limited.

## Figures and Tables

**Table 1:** Main drugs used in the pharmacotherapy of ET.

Drugs	Main Mechanism of Action	Therapeutic Effect	Main Side-Effects
First-line therapies Approved by the FDA, or supported by double-blinded, placebo controlled studies that meet the criteria for class I evidence (defined by USPSTF)			
Propranolol	Non-selective β-adrenergic receptor antagonist Anti-tremor mechanism: probably acting on peripheral non-cardiac β2 receptors (for more information see Section 2.4.1).	Tremor reduction by 50–70% (>50% ET patients respond), mainly for tremor affecting upper extremities, and head tremor response islimited	Frequent, rather mild, occurs in >60% ET patients: hypotension, bradycardia, slow heartbeat, fatigue, dizziness, exertional dyspnea, erectile dysfunction, headaches, somnolence
Primidone	Derivative of barbituric acid, antiepileptic drug Anti-tremor mechanism: still unknown; interacts with voltage-gated sodium channels or opening/potentiating GABA receptors	Tremor reduction by 50–70% (30–50% ET patients respond)	In 22–72% ET patients: sedation, fatigue, drowsiness, dizziness, ataxia, confusion, nausea, loss of coordination, anorexia, nausea, vomiting, flu-like symptoms
Second-line therapies Supported by double-blinded, placebo-controlled trials that do not meet the requirements for class I evidence studies			
Topiramate *Most effective second-line treatment*	Blockade of voltage-gated sodium channels, inhibition of high voltage-activated calcium channels Anti-tremor mechanism: not clear	Tremor improvement by 20–37% (30–40% response rate)	Paresthesias, difficulties with concentration, nausea, somnolence, fatigue, malaise, dyspepsia, weight loss, confusion, psychomotor slowing, abnormal taste perception, visual disturbances, nephrolithiasis Discontinuation of therapy because of adverse side-effects in approximately 30% of ET patients
Gabapentin Pregabalin	Structural analogs of GABA Inhibit α2δ subunits of voltage-gated calcium N-type channels; do not bind directly to GABA-A, GABA-B receptors Anti-tremor mechanism: not clear	Tremor improvement by 30–40% (approximately 30–50% response rate)	Sleepiness, dizziness, ataxia, nausea, weight gain in 30–40% of patients (mild) Discontinuation in cases of increased anxiety, insomnia, nausea, increased risk of depression and suicidal behavior
Alprazolam Clonazepam	Benzodiazepines Bind to GABA-A receptor complex, Cl^-^ influx	Tremor improvement by 30–50% (>50% response rate)	Sedation, cognitive impairment, tolerance, dependency, abuse, withdrawal symptoms, side-effects in approximately 50% of ET patients
Atenolol	Competitive β1-adenergic antagonist	Only in patients responding to propranolol (37% tremor reduction); response rate similar to other β-blockers	Similar to propranolol (see above), without possible bronchospasm
Metoprolol	Competitive β1-adenergic antagonist	Equal efficacy to propranolol (single dose), but no effect on tremor after chronic use	Similar to propranolol (see above)
Nadolol Sotalol Indenolol Arotinolol Timolol	Non-selective β-blockers	Effective, but only in ET patients who responded previously to propranolol	Most common side-effect isreduced alertness, otherwise similar to propranolol (see above) Adverse effects in approximately 25% patients
Third-line therapies Based on open-label studies or case series			
Nimodipine Flunarizine Nicardipine	Calcium channel blockers Binds to L-type voltage-gated Ca^2+^ channel	Tremor improvement by 50% (>50% response rate), based on studies on small numbers of ET patients	Most common: hypotension, oedema, headaches in 10–20% patients, dizziness, nausea, constipation, fatigue, palpitations and others (rather well-tolerated)
Clozapine	Atypical antipsychotic High affinity to D1, D4, 5HT2A, 5HT2C, 5HT6, 5HT7, α1- and α 2-adrenergic receptors, H1 and M1-M5 muscarinic receptors, weak affinity for D2 receptors	Tremor improvement by 50% (75% response rate), based on small clinical trials	Main: sedation, orthostatic hypotension, tachycardia, syncope, weight gain, metabolic syndrome (adverse effects in approximately 50% of ET patients) Possible: agranulocytosis, cardiomyopathy, tachycardia, risk of seizure, risk of neutropenia (not observed)
Olanzapine Quetiapine	Atypical antipsychotics High/moderate affinity to 5HT2A/C, 5HT6, 5HT7, D1-D4, H1, α1-adrenergic receptors, and M1-M5 muscarinic receptors	Tremor improvement by <50%	Insomnia, anxiety, headache, sedation, somnolence, dizziness, weight gain, orthostatic hypotension In long-term treatment there is an increased risk of parkinsonism

Division of drugs into first-, second-, and third-line therapies is based on Hedera et al. [79]. For more information see references [79,83,84]. Abbreviations: FDA: Food and Drug Administration, USPSTF: US Preventive Service Task Force.

**Table 2:** Examples of imaging studies with markers of dopaminergic system in patients with ET.

Imaging Study	Clinical Diagnosis, Subjects	Key Findings	References
Markers of DAT			
[123I]β-CIT SPECT	ET (n = 32), controls (n = 30)	No difference in the striatum between ET and controls	Asenbaum et al. (1998) [161]
[123I]FP-CIT SPECT	ET (n = 27), controls (n = 35)	No alterations in ET patients vs. controls	Benamer et al. (2000) [162]
	ET (n = 20), controls (n = 23)	No alterations in ET patients vs. controls	Isaias et al. (2010) [163]
[11C]FE-CIT PET	ET (n = 5), controls (n = 8)	No difference between ET patients and controls	Antonini et al. (2001) [164]
[123I]ioflupane SPECT	ET (n = 15), controls (n = 17)	No alterations in ET patients vs. controls	Di Giuda et al. (2012) [165]
	ET (n = 22), controls (n = 13)	No alterations in ET patients vs. controls	Waln et al. (2015) [166]
	ET (n = 12), controls (n = 10)	No alterations in ET with rest tremor vs. controls	Barbagallo et al. (2017) [167]
	ET (n = 28), controls (n = 28)	Mild striatal deficit in ET patients vs. control (less marked than in PD)	Gerasimou et al. (2012) [168]
	ET (n = 32, including 16 familial), controls (n = 31)	Mild striatal deficit in ET patients vs. control (less marked than in PD)	Isaias et al. (2008) [169]
[99mTc]TRODAT-1 SPECT	ET (n = 12), control (n = 10)	No alterations in ET patients vs. controls	Wang et al. (2005) [170]
[11C]dMP PET	ET (n = 6), controls (n = 10)	No alterations in ET patients vs. controls	Breit et al. (2006) [171]
Presynaptic radioligand (measures DA synthesis)			
[^18^F]DOPA PET	ET (n = 20, including 8 familial), controls (n = 30)	↓ 13% uptake in putamen (familial ET) and ↓ 10% (sporadic ET) vs. control	Brooks et al. (1992) [172]
Postsynaptic (D2 R)			
[123I]IBZM SPECT	ET (n = 11), no controls	No alterations in ET patients	Plotkin et al. (2005) [173]

DAT: dopamine transporter; ET: essential tremor.

## Data Availability

Not applicable.
